# Plant biosynthetic gene clusters in the context of metabolic evolution

**DOI:** 10.1039/d2np00005a

**Published:** 2022-04-20

**Authors:** Samuel J. Smit, Benjamin R. Lichman

**Affiliations:** Centre for Novel Agricultural Products, Department of Biology, University of York York YO10 5DD UK benjamin.lichman@york.ac.uk

## Abstract

Covering: up to 2022

Plants produce a wide range of structurally and biosynthetically diverse natural products to interact with their environment. These specialised metabolites typically evolve in limited taxonomic groups presumably in response to specific selective pressures. With the increasing availability of sequencing data, it has become apparent that in many cases the genes encoding biosynthetic enzymes for specialised metabolic pathways are not randomly distributed on the genome. Instead they are physically linked in structures such as arrays, pairs and clusters. The exact function of these clusters is debated. In this review we take a broad view of gene arrangement in plant specialised metabolism, examining types of structures and variation. We discuss the evolution of biosynthetic gene clusters in the wider context of metabolism, populations and epigenetics. Finally, we synthesise our observations to propose a new hypothesis for biosynthetic gene cluster formation in plants.

## Abstract 2

A significant subset of plant specialised metabolism genes are arranged in biosynthetic gene clusters. Here, we review these clusters and discuss their possible roles within the context of genome dynamics and metabolic evolution.

## Graphical abstract

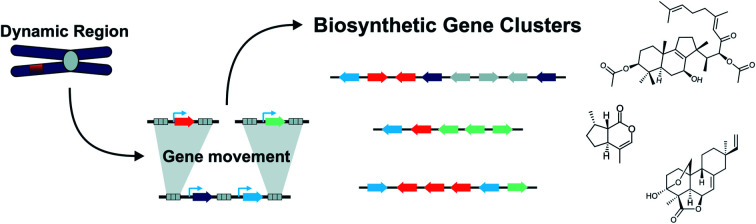

## 1 Introduction

Plants adapt to their environments by producing a range of complex chemicals that have roles including protection against herbivores,^1^ defence against pathogens,^2^ pollinator attraction,^3^ microbiome management,^4^ inter- and intra-plant signalling,^5^ and protection against oxidants.^6^ The role of plant specialised metabolism is well covered in recent reviews.^7^ The array of molecules produced are classified by structure and biosynthetic origin into groups including terpenoids,^8^ phenylpropanoids,^9^ alkaloids^10^ and glucosinolates.^11^ Many of these compounds are specialised metabolites, so-called because they appear in limited taxonomic range and may only be beneficial in specific ecological contexts. The ability of plants to modify and evolve new chemistry in response to changing environmental conditions may be a key part of their evolutionary strategy and success.^12^

As the environment continues to change, on both a local and global scale, plant chemistry will continue to adapt and evolve. What natural products we observe in plants today therefore constitutes only a snapshot of an ever-shifting mixture of molecules. It is within this context that we must consider the phenomenon of plant biosynthetic gene clusters (BGCs), tightly linked genomic regions that contain genes encoding the pathway enzymes for specialised metabolites. As the horizontal gene transfer that is responsible for tight linkage of biosynthetic genes in microbes is very rare in plants, these tightly linked genomic regions counter the classical view that gene location in eukaryotic genomes is largely random.

As more genomes are being sequenced, more and more BGCs are being discovered, and, rather than a curiosity, are now a core facet of plant specialised metabolism. It is now possible to predict BGCs computationally,^13,14^ an approach that can lead to the discovery of new plant metabolism.^15^

Plant BGCs have been reviewed multiple times.^16–23^ In this review we aim to examine BGCs primarily through an evolutionary lens. Crucially, we also examine structures closely related to BGCs including tandem arrays and gene pairs. We begin by describing and categorising genomic structures. Then we examine observed variation of conserved clusters both within and between species. These variations are the result of genomic rearrangements, the processes of which we examine in the next section. We then take a detailed look at the experimental examination of cluster evolution, including how clusters form and grow. Finally we look at the selective pressures that may be operating to form and maintain BGCs, and use these ideas to examine what function clusters may have. Through integrating ideas from population genetics and epigenetics, we propose a new hypothesis for BGC formation, which we hope will inform future research directions.

## 2 Genomic features of plant specialised metabolism

**Fig. 1:**
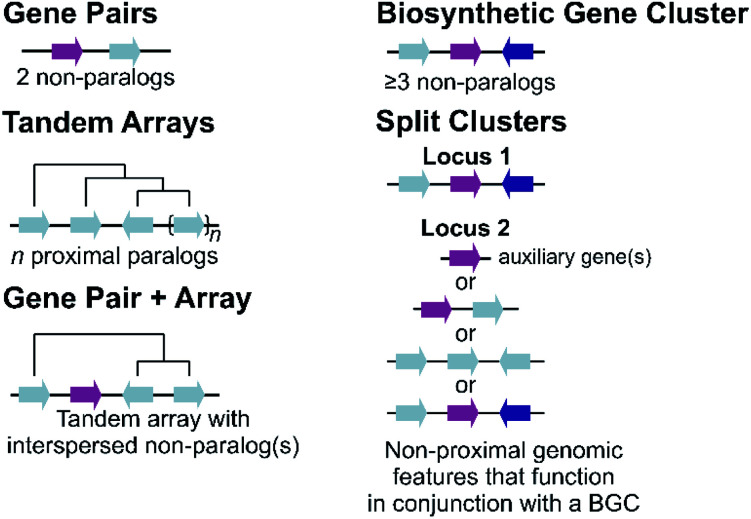
Genomic features of plant specialised metabolism. Non-paralogous genes are indicated by different coloured arrows with connecting lines indicative of a shared genomic region. Tree-like lines illustrate paralogous relationships.

Plant metabolic genes are organised in genomes in a number of different patterns. Such arrangements seem to reflect a continuum from a single randomly located gene to a complete clustered pathway: a journey from disorder to order. Tandem arrays, gene pairs and BGCs are key categories for describing gene organisation, but variations to these broad phenomena are common, including expanded gene pairs and split BGCs (Fig. 1). Genome wide analyses of metabolic gene organisation further highlights its complex and dynamic nature.

### 2.1 Duplications and arrays

The most simple gene arrangement compared to randomly located genes are tandem genes: two paralogous genes proximately positioned. One example are the cytochrome P450s (CYPs) CYP98A8 and CYP98A9 from Arabidopsis involved in the phenolamide pathway.^24^ More than two adjacent paralogs are classed as an array (Fig. 1). In rice, a three gene terpene synthase (TPS) array contributes to formation of diverse sesquiterpenes by producing different products.^25^ Large arrays of TPSs and CYPs, major contributors to metabolic diversity,^26^ are fairly common, with *Mentha* TPSs and Arabidopsis CYP71s, for example, appearing in tandem arrays of up to a dozen genes.^27,28^ Genes from multiple tandem arrays can interact to build layers of a metabolic network. In maize, three distinct tandem arrays (TPS, CYP71 and CYP81) interact to form a network of oxidised sesquiterpene antibiotics.^29^ Arrays can be linked to lineage specific metabolism, such as the CYP719 array in the *Coptis japonica* genome, which encodes enzymes catalysing multiple steps in the Ranunculales-specific protoberberine branch of the benzylisoquinoline alkaloids (BIAs).^30^ Transcription factors (TFs) involved in specialised metabolism are sometimes found in arrays, typically located apart from metabolic genes.^31,32^

### 2.2 Gene pairs

Gene pairs are adjacent metabolic genes with distinct evolutionary origins (*i.e.* not derived from recent duplications) (Fig. 1). Pairs containing TPSs are prevalent, especially TPS-CYP pairs. For example, the genome of lavender, a monoterpene producer, contains seven TPS-CYP and three TPS-ACT (acyltransferase) gene pairs.^33^ Conserved TPS-CYP gene pairs may underlie terpene diversification in eudicots.^34,35^ Analysis found non-random TPS-CYP associations in dicot genomes, with specific CYP families more commonly observed in pairs, including CYP71 and CYP72 with TPSs and CYP71 and CYP85 with oxidosqualene cyclases (OSCs).^34^ These associations were not found in monocots.

In a later-evolving example of TPS-CYP pairs, the Solanaceous species *Nicotiana* and *Capsicum* contain gene pairs encoding a TPS (5-*epi*-aristolochene synthase, EAS) and CYP (5-*epi*-aristolochene dihydroxylase, EAH), together responsible for the formation of the phytoalexin capsidiol.^36,37^*Nicotiana tobacum* has two EAS-EAH pairs,^37^ whilst *Capsicum annuum* contains three pairs controlled *via* a bidirectional promoter, in regions enriched more generally in EAS and EAH homologs.^36^ Such arrays of gene pairs represent a further level of complexity compared to a single gene pair.

The biosynthesis of diterpenoids can occur *via* two-step sequential activity of monofunctional class-II and class-I di-TPS.^38^ Gene pairs of these distinct TPS types are common, both alone,^39^ and as part of complex loci.^40–42^ These two TPS classes diverged prior to their association within extant genomes and so did not derive *via in situ* duplication. Another set of sequentially catalysed steps found in gene pairs are sester-TPSs coupled to prenyltransferases that are responsible for forming their unusual C25 substrates.^43^ These gene pairs are found across the Brassicaeae including in a three pair array in Arabidopsis.^44^

**Fig. 2:**
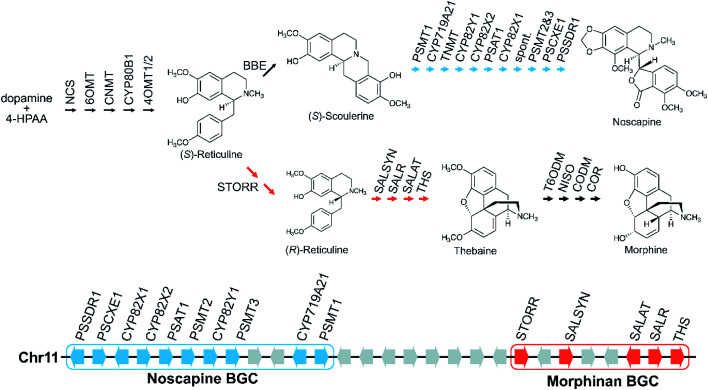
Genomic and biosynthetic origins of noscapine and morphinans in *Papaver somniferum*. Two BGCs on chromosome 11 are involved in the biosynthesis of noscapine (indicated in blue) and morphinans (indicated in red), respectively. Grey arrows on the chromosome represent genes that are not part of the pathways shown.

Gene fusions may be considered an extreme form of gene pairs: two genes have become so closely associated they share an encoded polypeptide chain.^45^ A notable example of this is the gene STORR (S-to-R-reticuline), which encodes a reticuline epimerase enzyme, a fusion of a CYP and alpha-keto-reductase (AKR) and is required for the formation of promorphinans in BIA biosynthesis (Fig. 2).^46,47^ Protein fusions are also observed in the tandem arrays of norcoclaurine synthase, also involved in BIA biosynthesis.^48^

### 2.3 Pairs and arrays

Tandem arrays which contain, or are proximal, to a non-homologous gene may alternatively be considered gene pairs with an expansion (Fig. 1). Whilst often referred to as clusters, these types of loci are strictly one non-homologous gene away from being a *bone fide* BGC. Reported examples are primarily TPS-CYP based and include a rice diterpenoid locus forming 5,10-diketo-casbene^49,50^ and a taxol-related biosynthetic locus in *Taxus*.^51^

### 2.4 Biosynthetic gene clusters

The conservative and robust definition of BGCs provided by Osbourn, which we employ in this review, is that a BGC must contain at least three genes of distinct evolutionary origin which contribute to a specific metabolic pathway (Fig. 1).^52^ This distinguishes a strict BGC from tandem arrays, gene pairs, and combinations of the two.

The idealised BGC contains genes that act sequentially (Fig. 1).^52^ The cyanogenic glucoside BGC of *Sorgum bicolor* has this form, with three adjacent genes from different origins (CYP79A1, CYP71E1 and UGT85B1) that together are sufficient to form the cyanogenic glucoside dhurrin from tyrosine.^53^

Large BGCs include those encoding noscapine biosynthesis in *Papaver somniferum*^54^ and avenacin biosynthesis in *Avena strigosa*.^55^ The noscapine cluster contains ten genes required to generate noscapine from the BIA precursor scoulerine^56^ (Fig. 2). The only gene missing, tetrahydroprotoberberine *N*-methyltransferase (TNMT), is also involved in a different pathway. The avenacin cluster is a complete 12-gene cluster, with 10 genes adjacent on a single scaffold, and two genes on a proximal scaffold that cannot be bridged due to repetitive elements.^55^ The avenacin and noscapine cluster appear to be arranged in an approximately “co-linear” manner, with the gene position reflecting the biosynthetic order.

### 2.5 Split clusters

**Fig. 3:**
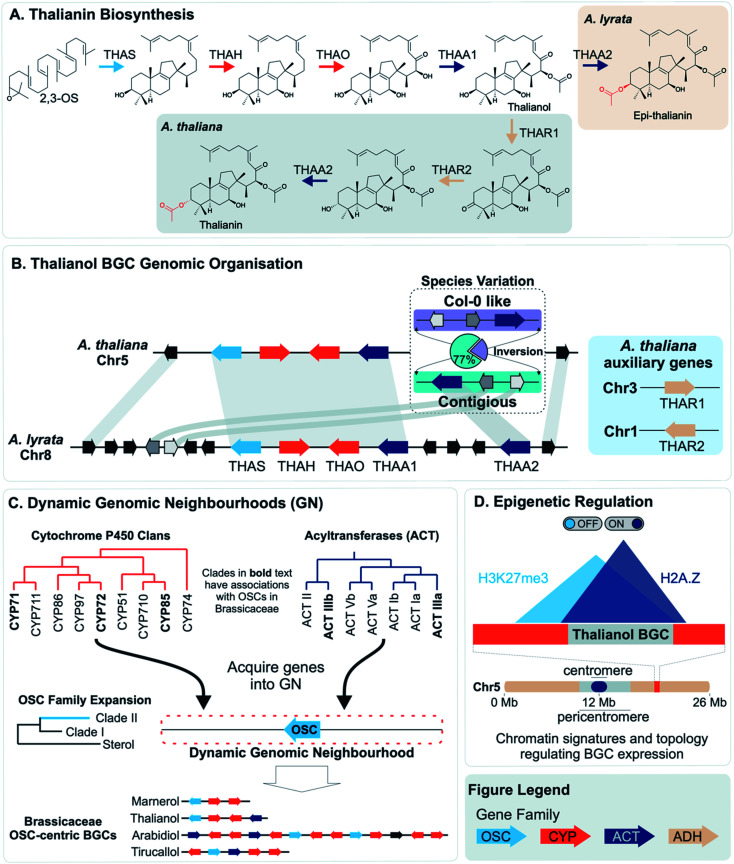
Graphical summary of Brassicaceae triterpene BGCs and the dynamic neighbourhood model for their evolution. (A) Thalianin biosynthetic pathway illustrating the difference between *Arabidopsis thaliana* and *A. lyrata*. (B) Genomic organisation and synteny of the thalianol BGC. The inversion observed in *A. thaliana* species is contrasted with the arrangement of genes for *A. lyrata.* (C) The movement of genes into dynamic neighbourhoods around clade II OSC members and known OSC-centric BGCs in Brassicaceae. (D) Chromatin signatures and overlapping topology involved in the activation or repression, respectively, of the thalianol BGC. The subtelomeric location of the BGC is also shown. Gene families shown in the figure legend are of the oxidosqualene cyclases (OSC), Cytochrome P450s (CYP), acyltransferases (ACT) and alcohol dehydrogenases (ADH). Gene arrows depict strand orientation with connecting lines indicating contiguous genomic regions.

More often than not plant BGCs do not fit their ideal, and it is common to find non-pathway intervening genes or intermediate steps catalysed by non-clustered genes. An example of these can be found in the paradigmatic (*epi*-)thalianin BGCs in *Arabidopsis lyrata* and *A. thaliana* (Fig. 3A and B).^57,58^ The orthologous BGCs contain an oxidosqualene cyclase (OSC), CYPs from two subfamilies and two acyltransferases (ACTs). However, in both species the clusters contain two intervening genes, and furthermore in *A. thaliana* two unclustered genes are involved in an epimerisation step (Fig. 3B).

**Fig. 4:**
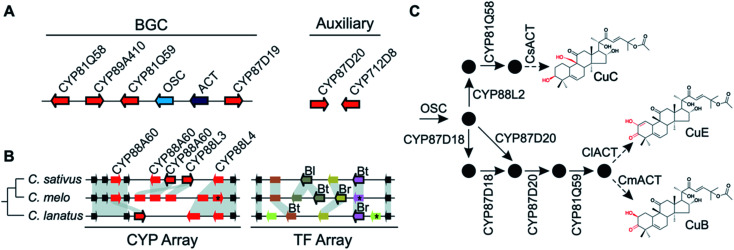
Cucurbitacin biosynthesis in Cucurbitaceae. (A) Cucurbaticin BGC and auxiliary genes conserved in *Cucumis sativus*, *C. melo* and *Citrullus lanatus*. (B) Syntenic relationships of tandem CYP and TF arrays showing species-specific genomic variations. Pseudogenes are shown as rectangles and genes disrupted by a premature stop codon are marked with an asterisk (*). Leaf- (Bl), fruit- (Bt) and root-specific (Br) TFs are indicated. Arrows with a black border indicate co-expressed genes predicted to contribute to biosynthesis of Cucurbatacins. (C) Biosynthetic pathway towards CuB, -C and -E with functionally characterised enzymes shown. Pathway intermediates are represented by black circles. The final step catalysed by the respective ACTs is preceded by an intermediate biosynthesised by a yet to be identified enzyme.

Pathways are often split across multiple genomic locations, such as a BGC interacting with unclustered tandem arrays (Fig. 1). An example of this is in cucurbitacin biosynthesis in Cucurbitaceae, where the pathway genes are found in a triterpenoid BGC cluster featuring OSCs, CYPs and ACTs as well as in an array of CYP88s on a different chromosome, a CYP subfamily with a role in gibberellin phytohormone metabolism (Fig. 4).^31,59^

Tanshinone biosynthesis in *Salvia miltiorrhiza* (Danshen) is split between a BGC, containing both classes of di-TPSs and CYP76s, and an array of CYP71s at a different location.^42^ In an example of a BGC-gene pair interaction, the large BGC on tomato chromosome 7 contains six pathway genes involved in steroidal alkaloid biosynthesis yet the key oxidation and transamination steps that incorporate the nitrogen into the triterpene scaffold are found together on chromosome 12.^60^

**Fig. 5:**
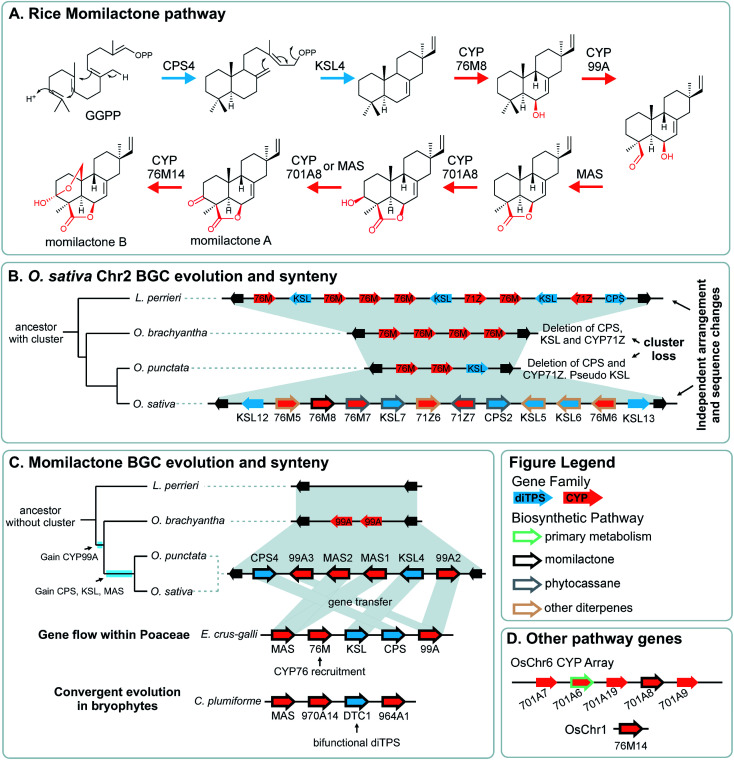
Graphical summary of momilactone BGC evolution and syntenic relationships. Note that the BGC is part of a split cluster with other loci involved in momilactone biosynthesis depicted in different panels. All genes involved in *O. sativa* momilactone biosynthesis are marked with a thick black outline. (A) Simplified pathway for biosynthesis of rice momilactones. (B) Structural differences and syntenic relationships relative to *Oryza sativa* chromosome 2 show independent evolution of the multifunctional phytocassane associated BGC. (C) Assembly of the momilactone BGC in different species showing gene gain events in *Oryza* spp., lateral gene flow to *Echinochloa crus-galli* and convergent evolution in bryophytes. (D) CYP array and auxiliary genes that are part of the momilactone pathway.

Pathway genes may also be found across multiple BGCs. The best characterised of these is the diterpenoid momilactone pathway in *Oryza sativa* which is split across two BGC regions in chromosome 2 and chromosome 4, a non-clustered gene on chromosome 1, and an array of CYP701s on chromosome 6 (a CYP subfamily with a role in gibberellin phytohormone metabolism) (Fig. 5).^61–70^ Furthermore, only the chromosome 4 BGC appears dedicated to momilactone biosynthesis, with the chromosome 2 region also responsible for the formation of other diterpenoids including the phytocassanes.

### 2.6 Genomes

Genome wide analyses have started to provide a view of BGCs that highlights their existence on a complex continuum from a single gene to tandem array to the co-regulated, polygenic, contiguous cluster exemplified by avenacin.^55^ In opium poppy, 70% of genes annotated as being involved in BIA biosynthesis are within 100 kb of other BIA genes,^71,72^ though these regions are mostly not BGCs but tandem arrays. There are also varying degrees of association, with some diffuse regions of many megabases (Mb) enriched in BIA genes existing alongside the tightly linked 584 kilobase (kb) BIA BGC containing genes from both noscapine and morphinan biosynthesis^71^ (Fig. 2). In a similar manner, the taxol-associated locus in *Taxus* is a 260 kb region that is within a 72 Mb region containing many other biosynthetic genes.^51^ Arabidopsis has three triterpene BGCs within a 5.3 Mb region on chromosome 5.^4^

Whole genome analysis of *Ophiorrhiza pumila*, a monoterpene indole alkaloid (MIA) producer, found 33 complex regions (*i.e.* arrays, pairs, clusters) associated with MIA genes but many did not show internal co-expression.^73^ MIA genes that do co-express are more likely to be in complex regions than those that do not co-express, but not necessarily the same regions as other co-expressing genes. This contrasts to features of a classically functional BGC where genes are co-regulated, sharing patterns of expression across different tissues and inductive conditions.^52^ This difference reflects both genomic complexity and the difference between robustly characterised and computationally predicted BGCs.^13^

There may be chromosomal regions more likely to contain clusters, even for different compounds. For example, the BIA BGC in poppy contains genes from two branching pathways (Fig. 2). In tomato an acylsugar associated BGC is adjacent to the large steroidal alkaloid BGC.^60,74^ Furthermore, chromosome structure may have a relationship with BGCs, with some clusters located close to the end of chromosomes, in subtelomeric regions.^34,55,75^

With more genomes available and biosynthetic pathways characterised, we are beginning to see that BGCs represent just one aspect of genome structure involved in metabolic complexity. The relationships between BGCs and structures such as gene pairs and tandem arrays are only now starting to be revealed.

### 2.7 Taxonomic distribution

The vast majority of characterised BGCs derive from the flowering plant lineage, angiosperms. Notable exceptions, described recently, are momilactone biosynthesis in *Calohypnum plumiforme*, a bryophyte Fig. 5C),^76^ and taxol biosynthesis in *Taxus* spp., of the Coniferophyte lineage.^51,77^ The lack of described examples outside angiosperms is not likely due to a real scarcity of BGCs in these lineages but rather due to the paucity of genome sequences, a result of genome complexity and investigation bias. As new genomes in these lineages are sequenced, more BGCs will be discovered.

The nature of BGCs in alga is less clear. Whole genome bioinformatics analyses has identified putative BGCs in green alga *Ostreococcus lucimarinus* and *Chlamydomonas reinhardtii*, as well as in the red algae *Cyanidioschyzon merolae*.^14^ A thorough analysis of chlorophyte genomes examined putative BGCs using three methods, optimised for bacterial, fungal or plant clusters.^78^ The plant-optimised method found no reasonable candidates, even in the chlorophytes (green alga), whereas the fungal and bacterial methods performed similarly well, finding an average of 5 clusters per chlorophyte genome. Many genomes were found to contain a type-I polyketide megasynthase, a multidomain enzyme common in bacteria and fungi, and unlike the smaller type-III systems found in plants. This gene has been partially characterised from *C. reinhardtii*.^79^

Based on current knowledge, chlorophyte BGCs appear to be more like bacteria or fungi than plants. The nature of clustering is likely to be related to growth and reproductive strategies, which varies greatly both between algal lineages and compared to plants.

## 3 Variation

Variation within a metabolically important genomic regions, such as BGCs, can be observed at both inter- and intra-species level. Observed differences include presence-absence variations (PAV), copy number variation (CNV) and larger genomic changes such as haplotype differences and chromosomal rearrangements. These differences illustrate the genomic structural flexibility and its contribution to biosynthetic variation.

### 3.1 Interspecies variation

Variation across species that produce different specialised metabolites can be reflected in genome organisation. For example, an array of TPSs in rice has PAV across species, accompanied by pseudogenes and neofunctionalised enzymes. The array content impacts their terpene chemotype and may contribute to species-specific ecological interactions.^25^

PAV of functional genes that impact plant chemotype is also observed for the thalianol and arabidin BGCs of *A. thaliana* and *A. lyrata*.^58^ The arabidin BGC of *A. thaliana* is absent in *A. lyrata*, indicative of larger genomic variations between species. As described above, the thalianol BGC is present in both species, but shows variation in the intervening non pathway genes (Fig. 3B). Furthermore, *A. thaliana* has specific co-expressing auxiliary genes that allow for an epimerisation that results in thalianin biosynthesis. Without these genes, *A. lyrata* forms *epi*-thalianin.

Variation in the genomic regions that encode for the cucurbitacin biosynthesis in cucumber (*Cucumis sativus* L.), melon (*Cucumis melo* L.) and watermelon (*Citrullus lanatus*) results in species-specific structural variations of cucurbitacins^31,80^ (Fig. 4). These three species share a conserved six-gene BGC but show species-specific variations in auxiliary genes, a CYP array and a TF array. The CYP and TF arrays feature species-specific duplications and pseudogenisation, which result in the chemotypic differences associated with the loss bitterness in domesticated varieties.

The exceptional quality of the *Ophiorrhiza pumila* genome allowed for identification of regions associated with MIA biosynthesis that have similar or differential arrangements in related species.^73^ The *O. pumilla* BGC containing strictosidine synthase, tyrosine decarboxylase and transporter, was syntenic with previously identified regions MIA producing species *Gelsemium sempervirens*^81^ and *Catharanthus roseus*.^82^ This is indicative of a conserved MIA BGC. In contrast, *Coffea canephora* lacks a strictosidine synthase in this region, which may account for the absence of MIAs in the species.

The aforementioned momilactone and phytocassane BGCs in *Oryza* show variation across species Fig. 5).^83^ Whilst the closely related wild relatives to *Oryza sativa* within the same AA genome lineage share both the chromosome 4 momilactone and chromosome 2 phytocassane BGCs, more distant *Oryza* lineages show variation. The momilactone BGC is absent in *O. brachyantha* and *Leersia perrieri*, but present in *O. punctata*Fig. 5C). In contrast, the phytocassane BGC is present in *L. perrieri*, but absent in *O. brachyantha* and *O. punctata*, where it is replaced by CYPs Fig. 5B).

### 3.2 Intraspecies variation

Intraspecies variation of plant specialised metabolites is proposed to enable rapid evolution in the context of changing environment.^84^ Examples of intraspecies variation within BGCs have been described. With the advent of long-read pan-genomes, we expect many more examples of such variation will be revealed.^85^

Short read re-sequencing of 1135 *A. thaliana* lines revealed a hierarchy of variation within the thalianol BGC, primarily benign single nucleotide polymorphisms and small indels in the gene UTRs.^58^ Gene deletions were observed in just 2% of accessions. Long-read comparative genomics of 22 of these accessions revealed a BGC inversion that results in compaction of the thalianol BGC relative to the col-0 accession (Fig. 3B). In the inverted clusters, THAA2 has moved into a contiguous arrangement with the four preceding genes of the BGC. Whilst this contiguous arrangement is observed for a majority of the accessions studied (17/22), phylogenetic analyses of the BGC variation does not group this compaction into a single clade. These results suggests either complex crossing of the locus between populations or multiple independent inversion events.

Opium poppy BIAs show intraspecies variation, with different varieties producing different types and amounts of alkaloids (Fig. 2). Noscapine biosynthetic genes were first identified through comparisons of high noscapine and low noscapine varieties.^56,86^ Crosses between varieties revealed the pathway was tightly linked, and subsequently sequencing revealed the noscapine component of the BIA BGC, which is absent in varieties that do not produce noscapine.^56,71^ Re-sequencing of multiple poppy cultivars has highlighted further CNVs and PAVs in metabolic genes.^72^

A genomic region containing TPS and CYPs is responsible for the formation of casbene-derived diterpenes in *Oryza*. The pathway was described in two independent studies, one using classic co-expression analysis,^50^ whereas the other leveraged 424 rice accessions and their metabolic diversity to conduct a metabolite-based genome-wide association study (mGWAS) and identify the biosynthetic locus.^49^ This gene cluster also exhibits haplotype variations where the intact cluster is observed largely in *O. sativa japonica* varieties with partial or absent clusters in *indica* varieties and *O. rufipogon*.

Examination of loci associated with zealexin biosynthesis across multiple maize genomes shows CNV as well as premature stop codons in some varieties, which impact genetic responses to elicitation and the consequent biosynthesis.^29^ Furthermore, lines generated from crossing producer B73 and non-producer Mo17 were used to map further loci involved in the biosynthetic pathway. Therefore, variations in both natural and artificial populations can be used to discover and understand biosynthesis and its genomic components.

Variation across species and populations highlights how the genome arrangement can reflect metabolic variation, with PAVs and variations in clusters correlating with chemotypes. Variation is also a useful tool for discovery, for example finding genes in different variants, but also in creating metabolite linked association studies. Finally, variation is crucial for understanding how BGCs evolved, as by looking at variants through a phylogenomic lens it is possible to infer the events that led to the formation, growth and loss of BGCs.

## 4 Genome rearrangements

Variation in the arrangement and order of metabolic genes on a genome is closely linked to metabolic diversity, as outlined above. This variation is mediated through a number of different genomic processes.

### 4.1 Gene duplications

**Fig. 6:**
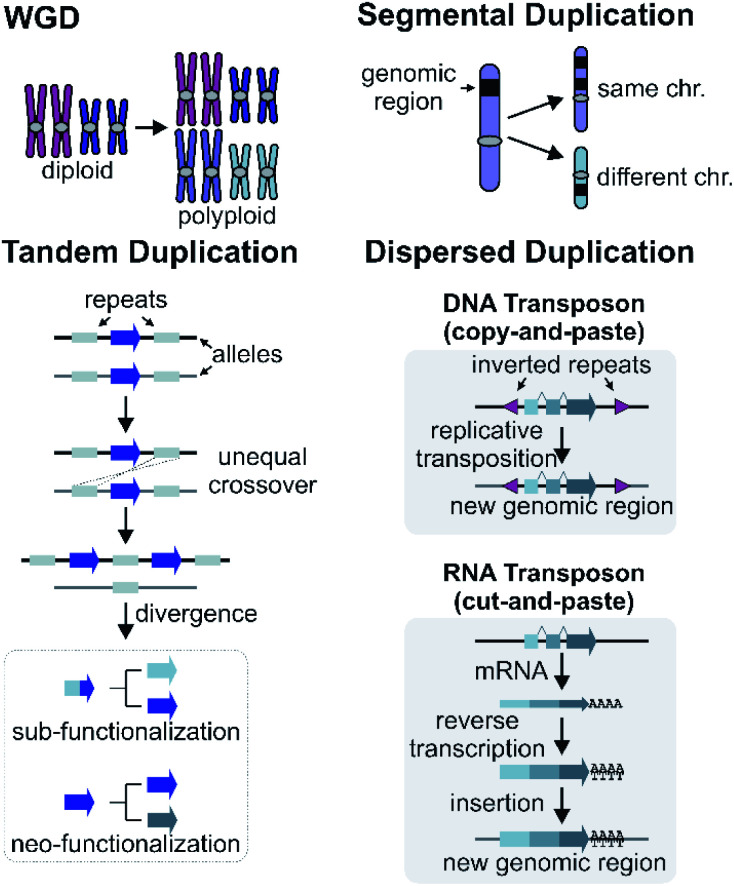
Mechanisms of gene duplications and genomic rearrangements.

Gene and genome duplication provide the raw genetic material for evolution (Fig. 6).^87–89^ Tandem duplication is thought to arise primarily through unequal crossing over (Fig. 6). This local duplication event gives rise to duplicates and arrays such as the rice or maize TPS arrays.^29,65^

Dispersed duplication results in a new gene copy being placed at a dispersed genomic region (Fig. 6). One mechanism for a dispersed duplication is replicative transposition, which is mediated by replication of type-II (DNA) transposable elements (TE) which may capture closely associated genes as they replicate.^90^ The second mechanism of dispersed duplication is through type-I (RNA) TEs (retrotransposons), which reverse transcribe mRNA and cause random insertion of intron-less gene copies in the genome.^91^ Ectopic recombination mediated by repeats (which may be TE derived) is a further possibility.^92^

In Arabidopsis, CYP98A3 underwent retroposition followed by tandem duplication to provide the two intronless CYP98A8 and CYP98A9. In lineages that preserve both genes, the paralogs have undergone subfunctionalisation to play specific roles in the phenolamide pathway.^24,93^

Dispersed duplications mediated either by type-I or type-II TEs are thought to contribute to gene recruitment into BGCs and other relevant regions. Analysis of BGC regions often highlights the presence of TEs, such as in the *Sorghum bicolor* cyanogenic glucoside gene cluster, *Oryza* hydroxycinnamoyl tyramine gene cluster,^94^ poppy noscapine cluster (Fig. 2)^56^ and thalianol/marneral gene clusters in Arabidopsis (Fig. 3).^95^

Thorough analysis of TEs in the opium poppy genome revealed that only in 5 of 18 regions associated with BIA metabolism there was an enrichment for specific TE classes.^72^ Furthermore, a subset of these TEs appeared to have been active relatively recently. Tandem duplicates also had associated TEs that were duplicated along with genes. Whilst TEs may have contributed to the evolution of BGCs and duplicates, the picture remains unclear and precise mechanisms and contributions of TEs are yet to be determined.

Enrichment analysis of TEs across multiple genomes revealed an increase in TEs proximal to genomic regions encoding for TPS-CYP pairs.^34^ Miniature inverted-repeat transposable elements (MITEs) were found to be related to blocks of gene pairs in eudicots. Furthermore, a correlation seems to exist between the chromosomal localisation of BGCs and regions where TEs are enriched.^35^

### 4.2 Segmental and whole genome duplications

Larger duplications, which can encompass multiple genes or sections of a chromosome, are termed segmental duplications (Fig. 6). There are likely to be multiple different sub-types of segmental duplication, based on size of duplicating region and mechanism of duplication, but details of these processes are not resolved. The poppy BIA BGC consisting of both noscapine and morphinan pathways is thought to be constructed through segmental duplications (Fig. 2).^71,96^

Whole genome duplication (WGD) constitutes the most drastic change of genetic material where the entire genome is doubled in the progeny (Fig. 6). WGD is often followed by rapid diploidisation in which duplicated essential genes are shed to restore genomic and biological stability.^97^ However, some duplicated genes (homeologs) can be retained.^98^

**Fig. 7:**
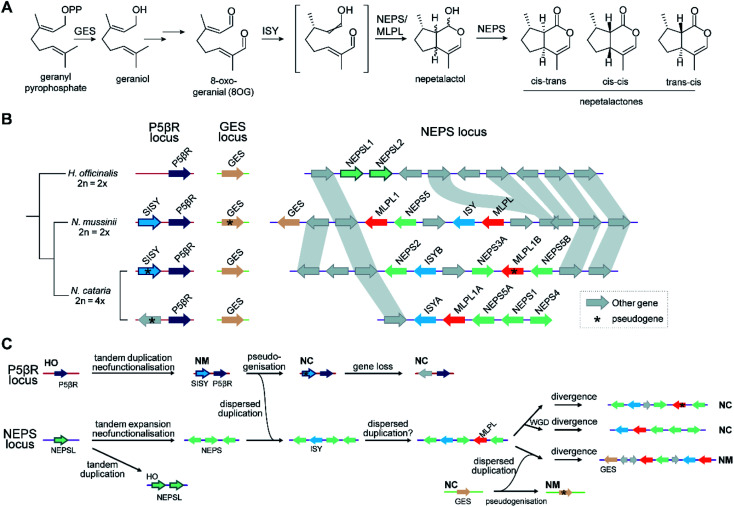
Nepetalactone biosynthesis in *Nepeta*. (A) Biosynthetic pathway for nepetalactone from geranyl pyrophosphate. *N. mussinii* (NM) and *N. cataria* (NC) produce three different nepetalactone stereoisomers as end products, controlled by NEPS (nepetalactol-related short chain dehydrogenase) paralogs and MLPL (major latex protein-like protein). (B) Genome sequences of *Hyssopus officinalis* (HO; non nepetalactone producer), *N. mussinii* and *N. cataria*, focussed on three loci of interest: P5βR, GES and NEPS. The P5βR (progesterone 5β-reductase) locus contains the iridoid synthase (ISY) paralogs P5βR and secondary-ISY (SISY). P5βRs have low but detectable ISY activity; *Nm*SISY has high ISY activity; *Nc*SISY is a pseudogene. The NEPS locus features the nepetalactone BGC containing NEPS paralogs, ISY and MLPLs. In *N. mussinii* it also contains a copy of GES. (C) Proposed chronology of nepetalactone BGC evolution based on biochemical and phylogenomic data. The initials next to a genome region shows it is found in an extant genome as shown in panel (B).

The new genes provided by WGDs can trigger the formation of new metabolism, as demonstrated in the diversification of triterpenoids in Maleae.^99^ WGDs of species with BGCs can lead to two paralogous BGCs, as seen in the tetraploid *Nepeta cataria*, which has two BGCs compared to the one in the diploid *Nepeta mussinii*^100^ (Fig. 7A and B). Similarly *Papaver setigerum* has two morphinan BGCs due to a WGD.^101^

The genomic disruption caused by a WGD in the *Papaver* lineage has been proposed to have triggered rearrangements (*i.e.* segmental duplications) that led to the formation of the BGC.^101^ However, with different species selected in the analysis, it appears that the segmental duplications may instead have preceded WGDs.^96^ This different interpretation highlights how evolutionary analysis is highly dependent on sample selection.

## 5 Cluster evolution

Putative steps in the origin, growth, variation and death of BGCs can be proposed by synthesising information from comparative genomics, phylogenetics and biochemistry. There are challenges to examining these timelines experimentally.^102,103^ However, with increased taxa sampling and increasingly sophisticated phylogenomic methods, we are beginning to piece together how BGCs emerged and diversified.

### 5.1 Tracking cluster origins

Attempts to understand the birth of a BGC may involve identifying the ancestral gene composition of relevant loci using comparative genomics, and predicting the activities of ancestral genes using phylogenetics, annotation or experimentation. The BGC responsible for nepetalactone biosynthesis in *Nepeta* was investigated using such an approach, including ancestral sequence reconstruction^100,103^ (Fig. 7). The study indicated that iridoid synthase (ISY), a key enzyme for nepetalactone biosynthesis, evolved from a tandemly duplicated ancestor prior to being recruited into a locus that contained an array of nepetalactol-related short chain dehydrogenases (NEPS), enzymes acting downstream of ISY^104^ (Fig. 7C). Another enzyme in the pathway, part of the major latex protein family, also moved into this NEPS locus. The now redundant tandem ISY is in a process of pseudogenisation, reflected by its erosion or loss in different syntenic regions. This timeline provides evidence for the evolution of enzyme activity prior to cluster formation, and the movement of genes between tandem arrays.

The *Oryza* momilactone metabolism emerged within the genus, and using comparative genomics broad steps in its evolution have been described^83^ (Fig. 5). The chromosome 4 momilactone BGC emerged first by addition of CYP99A to the syntenic locus, followed by recruitment of a class-I diTPS, a class-II diTPS and a dehydrogenase (Fig. 5C). The CYP76M genes in the chromosome 2 phytocassane BGC underwent duplications, both tandem and dispersed, to provide two new genes (CYP76M8 and CYP76M14) for the pathway (Fig. 5B and D).^63^ Finally, a CYP701A from a tandem array, that also contains CYP701A6 from primary metabolism, was recruited to catalyse the final step in the formation of momilactone B (Fig. 5D).^105^ Whilst the precise relative timing of the steps in momilactone enzyme and metabolic evolution are unresolved, the overall scheme highlights how multiple processes have contributed to genomic and enzymatic evolution of a new pathway.

The Solanaceae BGC involved in medium-chain length acylsugar biosynthesis also serves as an example of cluster formation.^74^ It is proposed to have formed around an ancestral BAHD acyltransferase gene with enoyl-CoA hydratase (ECH) and acyl-CoA synthetase (ACS) genes acquired later in a stepwise manner to form the cluster.^74^ The ancestral BAHD acyltransferase in the cluster is not active; instead a paralogous WGD duplicate (ASAT1) is involved in acylsugar biosynthesis. The acquisition of ECH is thought to have occurred before a Solanaceae-specific WGD event. The ACS gene likely moved into the BGC following a segmental duplication with the more parsimonious model of evolution supporting this movement after the WGD event. The BAHD, ACS and ECH genes all show Solanaceae specific tandem expansion at the BGC locus.

The gene STORR (S-to-R-reticuline), also named reticuline epimerase, is a fusion of a CYP and an alpha-keto-reductase (AKR) and is required for the formation of promorphinans in BIA biosynthesis (Fig. 2). It is a key part of a gene cluster and has been proposed to be the founding enzyme in its formation.^47,71^ Genome analysis shows that the genomic association of the separate CYP and AKR encoding genes predated the fusion event.^71,96,101^ However, a fused STORR has recently been identified in *P. californicum*, a plant that does not make (pro)morphinans, indicating it had a different role prior to the emergence of the morphinans.^96^ These results suggest that STORR may have been recruited from a different BIA branch to function in (pro)morphinan formation.

### 5.2 Broader principles of cluster assembly

In contrast to lineage specific recruitment of genes into BGCs, it has been proposed that, in eudicots, associations of TPSs with specific CYPs represent ancient “blocks” which are gene pairs that seed BGCs.^34^ These ancient combinations of CYPs and TPSs emerged between 90 and 130 Mya in the eudicot lineage, and are associated with MITE TEs which may help recruit genes or aid in co-expression of associated genes.^35^ Monocot terpenoid clusters do not have this deep synteny and instead may emerge *de novo* through a mix-and-match mechanism.

An alternative mechanism for cluster assembly and growth has been proposed based on triterpene biosynthesis in the Brassicaceae, centred around “dynamic genomic neighbourhoods”^106^ (Fig. 3C). These regions contain an OSC, and genes from a select set of triterpenoid related gene families (CYPs and ACTs) that have been recruited to these OSC regions. These regions, containing “mixed-and-matched” genes develop into BGCs through further recruitment, duplications and enzyme evolution. The BGCs forming *via* this process are superficially similar but independently assembled. Notably this proposal suggests clustering precedes the evolution of new metabolism.

### 5.3 Gene recruitment and cluster growth

Once established, BGCs and their metabolism may grow and diversify. For example, the aforementioned two step unclustered epimerisation in *A. thaliana* thalianol biosynthesis is absent in *A. lyrata* epi-thalianol biosynthesis (Fig. 3A and B). These two genes may have been recruited into the triterpenoid metabolism either after the divergence of *A. thaliana* and *A. lyrata* or prior to divergence (with *A. lyrata* experiencing loss of the genes). In either case, the fact they are not clustered supports a model of cluster expansion where enzyme activity evolution precedes clustering. Perhaps in the future, with increased selective pressure for thalianol biosynthesis, they will move into the thalianol BGC.

The recruitment of an active gene into a pre-existing cluster can be seen in *Nepeta*. The *N. mussinii* nepetalactone cluster contains geraniol synthase (GES), a TPS responsible for forming a nepetalactone precursor, but the gene is absent in *N. cataria* BGCs (Fig. 7B). In *N. mussinii*, the locus syntenic to the *N. cataria* GESs contains a pseudogenised GES. Thus, GES has been recruited to the nepetalactone cluster (Fig. 7C).

We previously described the BGC cluster of *Salvia miltiorrhiza* (CYP76s, CPs, KSKs) and its associated but separate CYP71D array, responsible for tanshinone biosynthesis.^42^ A syntenic TPS-CYP BGC is present in the related mint family species *Tectona grandis*, though this surprisingly has a contiguous CYP71D array.^107^ Without further analysis it is unclear whether the ancestral state of this BGC was with or without CYP71Ds; nevertheless, this highlights how genes, and even gene arrays, may be recruited, or lost, from BGCs.

Variation or loss of clustering across taxa can be seen with the DIMBOA pathway in the Poaceae, one of the first clustered pathways described.^108^ In maize, the cluster contains seven linked genes (Bx1-6) with another (Bx7) nearby. The cluster was proposed to have been established in the ancestral Poaceae initially by clustering of Bx1 and Bx2, followed by elongation with Bx3-5.^109^ In maize, the cluster has been maintained whereas in rye and wheat the Bx1-2 and Bx3-5 now form distinct clusters. As rye and wheat remain DIMBOA producers, this unclustering does not represent metabolism loss but perhaps different genomic organisation due to changes to linkage or regulatory requirements.

### 5.4 Gene flow

In a remarkable observation, a BGC encoding momilactone biosynthesis in *Oryza* was transferred to *Echinochloa crus-galli* presumably through hybridisation and introgression (Fig. 5C).^110,111^*Oryza* and *Echinochloa* are both in Poaceae but in different subfamilies: this BGC has undergone a gene flow between species.

The *E. crus-galli* BGC has gained a linked CYP76 gene, unclustered in known *Oryza* species. Phylogenetics indicates that the clustered TPSs and dehydrogenase in *E. crus-galli* are more closely related to *Oryza* than homologs from closely related species. The exception is CYP99A which appears more similar to related species perhaps indicating parallel evolution of this gene.

### 5.5 Cluster loss

Specialised metabolism is dynamic, and selection for specific metabolites is determined by environmental and ecological conditions, alongside the constant requirement for novelty in red-queen arms-races.^112^ As such, it would not be surprising for some metabolic pathways and their associated clusters to become under neutral or negative selection and be lost from genomes.

A limited number of examples of cluster loss have been observed. An intriguing example of cluster loss is observed in the genome of *Papaver setigerum*. Here the WGD duplicated morphinan cluster does not result in an increase in morphinans but instead one cluster copy shows erosion of *cis*-regulatory elements and loss of expression.^101^ However, this observation may be more related to subgenome dominance after WGD rather than cluster loss *per se*.

In *Oryza* diterpenoid biosynthesis, the chromosome 2 phytocassane associated BGC is present at the ancestor of the genus, but is lost in *O. punctata* and *O. brachyantha* (Fig. 5B). The CYP76M genes involved in the momilactone biosynthetic pathway remain present and active.^83^ Incorporating this observation with the birth of the momilactone BGC described above, we gain a picture of dynamic diterpenoid metabolism in the *O. punctata* and *O. brachyantha* lineages where the ancestral phytocassane BGC and its associated pathways may be becoming co-opted and superseded by a new momilactone metabolism.

### 5.6 Convergence

As the number of examples of BGCs and related features in plant genomes increase, there is a corresponding increase in the observation of genomic convergence, where biosynthetic loci with similar compositions, leading to similar or identical products, are found in phylogenetically separated species where the common ancestor lacks such a cluster. Casbene derived diterpenoids, for example, are found in both *Euphorbaceae* (dicot) and *Oryza* (monocot), and their formation in both taxa are controlled by loci containing TPSs and duplicated CYPs.^49,50,113^ Both the genes and the genomic structure involved in the pathway evolved independently.

A locus for cyanogenic glucoside biosynthesis has arisen independently in *Lotus japonica*, cassava (*Manihot esculenta*) and sorghum (*Sorghum bicolor*).^53^ The pathway in all species involves two sequential CYP steps and a glucosyltransferase. Although the pathways are independently assembled, the CYP and UDP-glucosyltransferase (UGT) subfamilies that contribute are similar, with CYP79s and UGT85s contributing to the oximine formation and glycosylation in all species. Thus, the pathway evolution was indeed independent but also somewhat parallel as the enzymatic starting point was similar.^114^

A striking example of convergence is the evolution of momilactone biosynthesis and a corresponding cluster in both *Oryza*, a genus in Poaceae (grasses), and *Calohypnum plumiforme*, a bryophyte (Fig. 5C).^76^ Like the *Oryza* BGC, the *C. plumiforme* cluster contains TPS, CYPs and a dehydrogenase. Notable differences are the presence of two CYPs in the *C. plumiforme* pathway, compared to three in *Oryza*, and a single bifunctional diTPS instead of the two monofunctional diTPSs in *Oryza*. Thus, the TPS gene subfamilies are different, plus no synteny is apparent across species, emphasising the that pathways are convergently evolved.

The convergence of metabolism does not always lead to convergence of genomic structure. A number of lineages in the Caryophyllales evolved betalain biosynthesis in parallel, through recurrent evolution of the enzyme l-DOPA 4,5-dioxygenase (DODA).^115^ In the Amaranthaceae acquisition, DODA and CYP76AD1 form a gene pair, conserved in multiple betalain producing species. *Mesembryanthemum crystallinum* acquired betalain biosynthesis in parallel, within the Aizoaceae lineage, but its DODA and CYP76AD1 enzymes are unpaired and on different chromosomes.

The concept of dynamic genomic neighbourhoods provides a more subtle view of convergence and divergence in which similar BGCs are independently assembled and can lead to different products (Fig. 3C).^106^ Superficial similarities between BGCs lie in the “shuffling” of a conserved set of genes related to a compound class. This is observed when comparing the *Capsella rubella* tirucallol cluster with the Arabidopsis thalianol cluster, as well as in a seemingly non-functional BGC in *Brassica rapa*. All BGCs contain OSCs, CYP708s and CYP705s but the genes do not appear to be true orthologs and furthermore are located in different karyotype blocks indicating independent assembly.

## 6 Cluster selection and function

The proximate origins and properties of BGCs have been extensively discussed above. However, these features must emerge through some more ultimate cause: selection. Evolutionary pressure must drive formation and maintenance of BGCs, perhaps indicating BGCs may have some specific functions in specialised metabolism.

BGCs and metabolism are closely related and any selection for a BGC must be linked to selection for specific specialised metabolites. Specialised compounds are often involved in interactions with other organisms, for example in defence or symbiosis, which provide a selective advantages.^116^ Roles for specialised metabolites produced by BGCs include modulation of microbiomes,^4^ anti-bacterial or anti-fungal phytoalexins^36,117^ and insect interactions.^100,118^ A subset of specialised metabolites encoded by BGCs appear to have become more integrated into physiological processes, with roles in root growth^4^ and drought resistance (stomatal opening).^119^

### 6.1 Enzyme and metabolic evolution

BGC evolution is tied to enzyme and metabolic evolution. The classic model for enzyme evolution is of gene duplication leading to neo/subfunctionalisation and the emergence of new enzyme activities. Initial maintenance of duplicates may be driven by gene dosage, as indicated in analysis of the poppy genome.^72,88^ Promiscuous enzymes can have low activity initially toward a new substrate which can be selected for during pathway formation.^114,120^

The evolution of new metabolism requires multiple new enzymes to evolve. There are a number of proposed models for this process, recently synthesised into a metabolite-enzyme coevolution model.^121^ Promiscuous enzymes generate low abundance side products which serve as the starting point for evolution (an “underground” metabolism).^122,123^ Once a compound is under selection, rate determining steps in its pathway emerge first, with other steps appearing sequentially. This compelling model does not require selection for all intermediate compounds, nor does it require the simultaneous recruitment of multiple genes.^121^

In plant systems, there are other considerations. Recruitment of genes and chemical precursors from primary metabolism is a common occurrence in the formation of metabolites.^122,124^ In plants, co-regulation across space and time is an important feature of functional pathways, due to their sessile nature and anatomical/morphological plasticity. Shoji's recruitment model of plant metabolic evolution proposes that the recruitment of promiscuous enzymes into pre-existing regulons through promoter evolution is a major force in developing new metabolism.^125^

### 6.2 Co-regulation

Broadly, there are two viewpoints on cluster function: the co-regulation hypothesis and the co-inheritance hypothesis. The co-regulation hypothesis focuses on the functional advantages of being in a cluster, whereas the co-inheritance hypothesis centres on the impact of linkage itself on an evolving population. A combination of these two factors may be at play, however, for clarity, we address them as exclusive theories.

The co-regulation hypothesis posits that clustering somehow aids in the co-regulation of genes in a pathway. Functional metabolic pathways typically share expression patterns across multiple tissues and may be induced by similar triggers. The role of BGCs in co-regulation could come in two forms: the functional view that clustered genes in BGCs can be more tightly regulated than unlinked genes, or an evolutionary view that clustering can expedite recruitment of genes into the same regulon during metabolic evolution.

Whilst many BGCs, especially those that are well characterised, show tight co-expression,^55^ it is also clear that unclustered genes can also co-express well with those in gene clusters. This is seen in individual examples of split BGCs,^63^ as well as in whole genome analyses of clustered pathway genes.^73^ Of course, unclustered metabolic pathways are also co-regulated, and co-regulation is a better predictor than gene proximity in determining functional cooperativity.^126^ These observations raise a challenge to a purely functional co-regulation viewpoint.

However, it is possible that clustering may aid in the rapid recruitment of genes into a regulon. There is evidence that BGCs have specific epigenetic properties which may aid their co-regulation (*e.g.* H3K27me3, H2A.Z) (Fig. 3D).^49,127^ Some BGCs also appear to compartmentalise in three dimensions, interactions also related to these epigenetic markers^128^ (Fig. 3D). The epigenomic aspects of clustering may provide shortcuts into gene regulon recruitment, providing fuel for the recruitment model of plant metabolic evolution^125^ and an evolutionary co-regulation view of BGC formation.

### 6.3 Co-inheritance

The co-inheritance hypothesis centres on genetic linkage: by clustering genes, the chances of inheriting a whole intact pathway are maximised as recombination breakpoints between genes becomes more unlikely the closer they are linked. This phenomenon can operate when inheriting a whole pathway is much more advantageous than inheriting a partial pathway. This occurs if only the final end-point product provides a selective advantage, and also when intermediates in the pathway are toxic.^111^ Intermediate toxicity has been proposed to account for the organisation of genes in certain large clusters which appear to be co-linear with respect to the biosynthesis (*e.g.*Fig. 2).^55,56^ Genes at the end of the clusters are at greatest risk of loss (especially in subtelomeric clusters), and so enzymes that form toxic products would be at the cluster termini. Alternatively co-linearity may reflect the order of recruitment into a cluster. Interestingly, the co-inheritance hypothesis disfavours stepwise metabolic evolution where each pathway step must be under selection sequentially.

The co-inheritance argument is weakened by the presence of incomplete or split clusters: do all pathway steps not need to be inherited together? Some unclustered genes may be linked closely to a paralogous vital gene, such as the CYP701A8 involved in momilactone biosynthesis which is close to CYP701A6: kaurene oxidase from gibberellic acid metabolism (Fig. 5D).^111^ It has been proposed that this arrangement aids in the inheritance of the specialised metabolism gene, negating any co-inheritance advantage for it to be in a BGC.^129^ Whilst without linkage to the functionally related BGC there will not be enhanced likelihood of whole pathway co-inheritance, if the gene has roles in multiple pathways, clustering may in fact be disadvantageous. Incomplete or split clusters do not negate the co-inheritance argument but just highlight that the extant BGCs are in a dynamic state.

The co-regulation and co-inheritance hypotheses may predict different sequences of steps in the interplay between enzyme, metabolism and cluster evolution. The co-regulation hypothesis implies metabolism formation (and enzyme evolution) occurs largely after genes are clustered and whilst they are being recruited into regulons, whereas the co-inheritance hypothesis requires selection of an existing pathway (which requires active enzymes) to drive linkage. Thus, tracking the relative timing of enzyme evolution/recruitment and cluster formation may be able to distinguish between the two.

As pathway evolution must precede clustering in the co-inheritance hypothesis, we would expect functional unclustered genes to be driven into clusters over time, assuming the pathway is under selection. Investigations of *Nepeta* BGC formation^100^ (Fig. 7), as well as other observations of gene recruitment into active clusters (section 5.3) seem to support this chronology, lending weight to the co-inheritance hypothesis.

### 6.4 Evolutionary playgrounds

An emerging phenomenon are dynamic genomic neighbourhoods, sometimes styled as an ‘evolutionary playgrounds’, primarily described in the Brassicaceae (Fig. 3C).^22,95,106^ These are genomic regions that are enriched in a core set of genes related to specialised metabolism. Whilst often these may encode a single BGC, in some cases they may not necessarily be associated with a single co-regulated functional pathway.

We note that similar complex BGC-like regions have been described outside the Brassicaceae. For example, the chromosome 2 BGC in *Oryza sativa* contains multiple tandemly duplicated genes with diverging functions that contribute to multiple pathways (Fig. 5B).^117^ A complex locus for terpene biosynthesis is found in *Solanum*, containing TPSs from different families alongside *cis*-prenyltransferases (CPTs).^40^ The region generally appears to contribute to terpene diversity derived from unusual precursors but does not clearly target a specific end-product.

In the co-regulation model BGC-like genomic neighbourhoods may represent premature BGCs undergoing recruitment into regulons, whereas in the co-inheritance model, they more likely represent older BGCs which originally contributed to a single pathway but after internal duplications, recruitment and divergence have diversified. The regions may be dynamic, recruiting and duplicating genes that may gain new roles in multiple metabolic pathways;^95^ they may contribute more to general metabolic diversity rather than to the accumulation of a specific metabolite.

Dynamic genomic processes have left genomes with features that appear to be BGCs based on gene annotation and proximity, but they do not demonstrate co-expression or functional cooperation, the latter being a necessary part of a typical definition of BGCs (see section 2.4).^52^ These pseudo-BGCs are often identified by genome wide plant BGC identification algorithms when searches are not constrained by co-expression.^126^ These features should not be conflated with *bone fide* BGCs but instead may represent dynamic genomic neighbourhoods or perhaps BGCs in the process of formation or erosion.

### 6.5 Genome architecture and TEs

Genomic neighbourhoods, whether they contain a complete BGC or not, may have features that make formation of BGCs favoured. Firstly, they tend to be located within dynamic chromosomal regions enriched in transposable elements, such as subtelomeric regions^55,75,108^ or between WGD boundaries.^95^ These regions may be enhanced in segmental duplications and rearrangements, as well as acceptor sites for dispersed duplications.^130^ These regions may share chromatin states and associated epigenetic markers that could facilitate coordinate regulation of gene expression.^127,128,131^ Furthermore, tandem duplicates demonstrate a greater frequency of dispersed duplications due to association with flanking repeats.^132^ This may lead to exchange between tandem arrays and dynamic genomic neighbourhoods.

As described above, the histone marker H3K27me3, associated with transcriptional repression, has been shown to be associated with BGCs and has been evoked as a facet of intra-cluster regulation (*e.g.*Fig. 3D).^127,128^ In plants, this marker has also been found to be involved in long range genome interactions and chromatin clustering,^133,134^ and is a key feature of co-regulation of distant genes.^135^ Furthermore, it is potentially associated with tandem repeats^136^ and topologically associated domains (TADs)^137^ which have been found to have high recombination rates potentially allowing accumulations of variants.^138^ There is also a link to TEs: H3K27me3 associated recombination hotspots in rice are associated with MITEs,^139^ and the active Arabidopsis TEs ATENSPM3 and ATCOPIA93 preferentially target genes with H3K27me3 and H2A.Z,^92^ a marker also associated with BGCs.^127^ H3K27me3, along with H3K18ac, is involved in induction of biosynthetic genes to pathogen responses in Arabidopsis.^140^

It appears that H3K27me3 may be involved not just in intra-cluster regulation but in the co-regulation and spatial connection of genetically distant loci involved in the same pathway. Perhaps through H3K27me3, loci that are unclustered on the linear genome can cluster in three dimensions.

### 6.6 Linkage and populations

The presence of adaptively or functionally related genes clustered together in eukaryotes is not unique to plant specialised metabolism. In fact, similar regions to BGCs known as genomic islands or supergenes are known for other traits in plants and animals.^141–143^ There are emerging theories regarding these genomic islands that may provide insight into plant BGC formation and selection.

A population genetics model indicates that “concentrated genetic architectures” (*i.e.* BGCs) can emerge when a locally adaptive trait is evolving within a wider population, and there is migration between the populations.^144^ In this scenario, clustering maintains a polygenic trait through tight linkage: the stronger the selection for the adaptive trait, the larger the cluster can be. In contrast, a globally adaptive trait does not lead to clustering. In this model, intraspecies variation, and gene flow between these populations, is a necessary aspect of cluster emergence and maintenance. A cluster will decay if it becomes ubiquitous in a population.

This may account for which type of pathways are in clusters. Older ubiquitous multistep specialised metabolic pathways such as core phenylpropanoid biosynthesis are typically not found in clusters. Notably, the late stage branching pathways in opium poppy BIA biosynthesis are in BGCs whereas the earlier steps are not^71^ (Fig. 2). This correlates with the fact that alkaloids are ubiquitous in the species, but the downstream branching pathways like noscapine demonstrate intraspecies variation.^56,72^

This local adaptation–migration model may even account for cluster variation and recruitment: once a trait is established, modifications and fine-tuning of the trait may occur through recruitment and competition of linked alleles.^145^ This is has parallels to a BGC centred on a founding enzyme (*e.g.* TPS/OSC) with varying tailoring enzymes (*e.g.* CYPs, ADHs, ACTs) (*e.g.*Fig. 3C).^106^

However, further modelling by Yeaman has led to the surprising observation that the standard theory of co-inheritance, where linkage is favoured as recombination of a polygenic trait is repressed, may not be sufficient to account for clustering.^146^ This is because, assuming random distribution across a genome, the nascent cluster is a small target for a translocating gene, and off-target translocations will dominate. Instead, processes that target co-adapted loci for rearrangements are required. This suggests that cluster formation, prior to selection for the BGC, is not random but under some control, potentially shaped by natural selection of cluster forming processes.^146^ This could indicate clusters have a specific adaptive advantage, as suggested by the co-regulation hypothesis. Alternatively, it is proposed that spatial proximity of unlinked but similarly adapted alleles may lead to their rearrangement into clusters.^146^

Although speculative, this theory has parallels in plant BGCs and epigenetics discussed above. H3K27me3 mediated long-range chromosomal interactions may bring co-regulated metabolic genes together in space. This physical proximity may facilitate the exchange of genetic material between loci, mediated by repeats or TEs.^147^ In this manner BGCs can be built.

### 6.7 Hypothesis for BGC formation

**Fig. 8:**
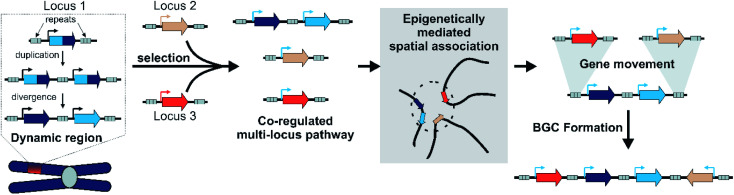
Model for plant BGC formation.

By integrating models of local adaptation,^144–146^ with modern concepts in plant specialised metabolism evolution,^8,114,120–122,125^ emerging descriptions of plant genomic architecture,^134,137,138^ and BGC evolution studies,^74,83,100,106^ we propose a new hypothesis to account for BGC formation and growth (Fig. 8).

We accept this is highly speculative but hope it can inform future avenues for investigation. Crucially, we expect the following process to occur in a subpopulation under local selective pressures within a larger population. We predict that certain gene family types associated with locally adaptive traits may preferentially associate with dynamic genomic regions. These genes may undergo tandem expansions, initially fixed by gene dosage. Their promiscuous activities generate an underground metabolism, of which some compounds may be adaptive. Under selective pressure for certain compounds, unlinked genes will subfunctionalise through specialisation gaining enhanced specific activities and modifications to promoters and expression. A new pathway is formed.

These newly co-regulated genes share epigenetic signatures (*i.e.* H3K27me3) and associate in three dimensional space. This physical proximity increases the chances of genes moving between adaptive loci, mediated by TEs either through ectopic recombination or active transposition. Thus pathway genes are rearranged into clusters, which are maintained primarily as they allow the inheritance of a complete polygenic adaptive trait. In these new clusters other genes encoding tailoring enzymes may be recruited or lost to fine-tune the active compound. Clusters will decay if the biosynthesis becomes globally adaptive in the population, or if new compounds provide greater advantage.

## 7 Conclusion

Investigation into the structure, function and formation of plant BGCs is a compelling interdisciplinary pursuit. It sits at the interface of multiple fields: plant biology, genomics, evolutionary biochemistry and biosynthesis. Emerging research has the potential to encompass further diversity including *epi*-, phylo- and pan-genomics; chemical ecology; and population genetics. Our understanding of plant BGCs will be influenced by developments in these wider fields, however, the unique combination of phenomena at play in plant BGCs will also lead to novel biological insights with wider impact. The study of plant BGCs show us how plants use a genetic toolkit to rapidly form new chemistries to help them thrive in a changing world.

## 8 Conflicts of interest

There are no conflicts to declare.

## Supplementary Material
